# Type 1 Diabetes Mellitus: Cellular and Molecular Pathophysiology at A Glance

**DOI:** 10.22074/cellj.2018.5513

**Published:** 2018-05-28

**Authors:** Bahar Saberzadeh-Ardestani, Razieh Karamzadeh, Mohsen Basiri, Ensiyeh Hajizadeh-Saffar, Aisan Farhadi, A.M. James Shapiro, Yaser Tahamtani, Hossein Baharvand

**Affiliations:** 1 Department of Stem Cells and Developmental Biology, Cell Science Research Center, Royan Institute for Stem Cell Biology and Technology, ACECR, Tehran, Iran; 2 Clinical Islet Transplant Program and Department of Surgery, University of Alberta, Edmonton, AB, Canada; 3 Department of Developmental Biology, University of Science and Culture, Tehran, Iran

**Keywords:** Diabetes Complication, Environment, Etiology, Genetic, Type 1 Diabetes Mellitus

## Abstract

Type 1 diabetes mellitus (T1DM) is a disease where destruction of the insulin producing pancreatic beta-cells leads to increased blood sugar levels. Both genetic and environmental factors play a part in the development of T1DM. Currently, numerous loci are specified to be the responsible genetic factors for T1DM; however, the mechanisms of only a few of these genes are known. Although several environmental factors are presumed responsible for progression of T1DM, to date, most of their mechanisms remain undiscovered. After several years of hyperglycemia, late onsets of macrovascular (e.g., cardiovascular) and microvascular (e.g., neurological, ophthalmological, and renal) complications may occur. This review and accompanying figures provides an overview of the etiological factors for T1DM, its pathogenesis at the cellular level, and attributed complications.

## Introduction

Type 1 diabetes mellitus (T1DM) is an autoimmune disease that results from beta-cell destruction in pancreatic islets. Although it may occur at any age, T1DM most typically presents in adolescence with a peak onset around puberty. The incidence of T1DM is equal in both sexes during childhood, but males more commonly present with this disease in early adult life (1). Although previously most prevalent in Europeans, it is becoming more common in other ethnic groups. The International Diabetes Federation (IDF) 2015 Atlas has estimated that 415 million people worldwide have diabetes. This number is predicted to increase to 642 million by 2040 (2). T1DM comprises 5-10% of all causes of diabetes and is one of the most frequent autoimmune diseases of early life. The incidence of T1DM is escalating in all populations. It has been predicted that the incidence of T1DM in the under 5-year-old age group will increase two-fold in less than 20 years in Europe (3). 

Although the precise causes of T1DM remain unknown, it is clear that both genetic and environmental factors play a role. The genetic region most strongly linked to T1DM is the human leukocyte antigen (HLA) locus (4). However, not all diabetes-related genetic factors are related to the immune system since genes associated with insulin production or beta-cell function have also been identified. Environmental factors are important in T1DM to the extent that monozygotic (identical) twins with identical genomes may have different health fates due to exposure to different environmental factors (4). In contrast to the tremendous amount of data about the role of genetic factors in T1DM pathogenesis, there is much less information about the role of environmental factors. Because of the complexity of environmental parameters, their mechanisms of action are mostly unknown (5). 

**Fig.1:**
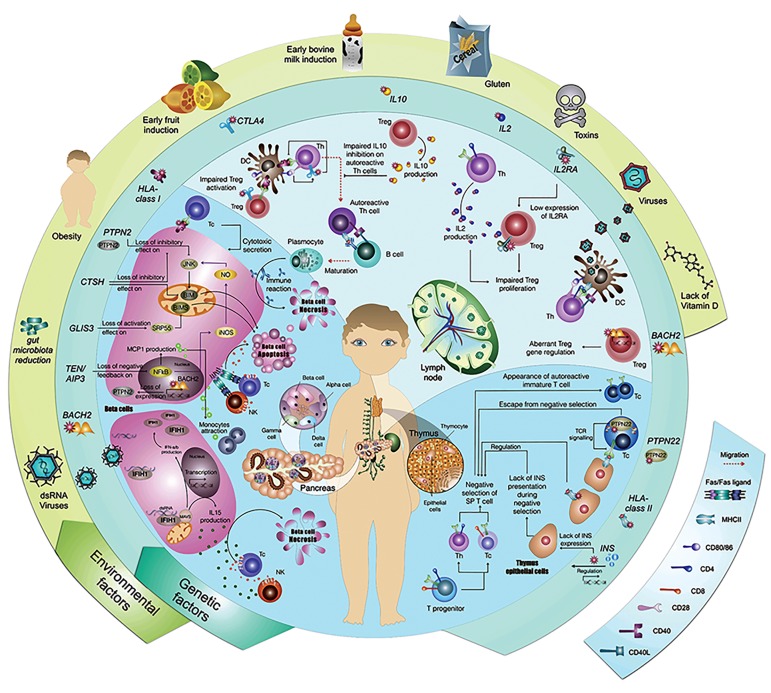
Genetic, immunologic, and environmental etiologies of type 1 diabetes mellitus (T1DM). The outer circle shows some of the most important environmental etiologies of T1DM and the inner circle presents some of the most important genetic etiologies. The central circle demonstrates each genetic or environmental factor’s known mechanisms of action. The left lower part of the circle shows the dsRNA virus, TEN/AIPS, GLIS3, CTSH, PTPN2 and HLA class 1 mechanism of action at the cellular level in the pancreas microenvironment, which leads to either necrosis or apoptosis of islet beta-cells. The upper part of the circle shows CTLA4, IL10, IL2, IL2RA, BACH2, and viral mechanisms of action in the lymph node. The right lower part of the circle shows PTPN22, HLA class2, and insulin mechanisms of actions which take place in the thymus. AIP3; Actin interacting protein 3, CTLA4; Cytotoxic T-lymphocyte associated protein 4, CTSH; Cathepsin H, GLIS3; GLIS family zinc finger 3, HLA; Human leukocyte antigen, IFIH1; Interferon induced with helicase C domain 1, IL; Interleukin, IL2RA; Interleukin 2 receptor subunit alpha, INS; Insulin, JNK; c-Jun N-terminal kinase, MAVS; Mitochondrial antiviral-signaling, PTPN2; Protein tyrosine phosphatase non-receptor type 2, PTPN22; Protein tyrosine phosphatase non-receptor type 22, BACH2; BTB domain and CNC homolog 2, Tc; Cytotoxic T cell, Th; Helper T cell, NK; Natural killer cell, Treg; Regulatory T cell, DC; Dendritic cell, SP T cell; Single positive T cell, TCR; T cell receptor, and NF-.B; Nuclear factor kappa-light-chain-enhancer of activated B cells.

**Fig.2:**
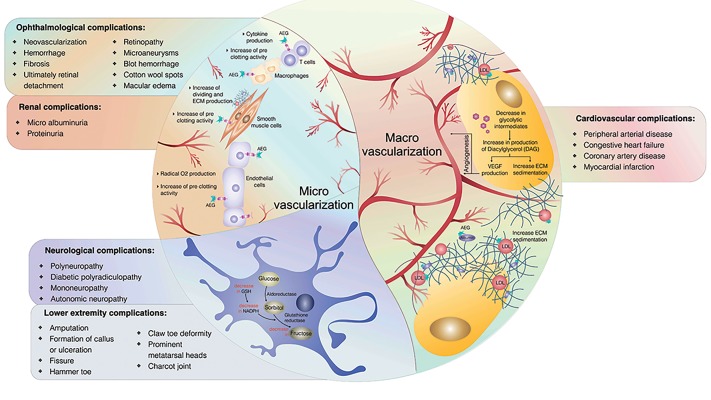
Chronic complications of type 1 diabetes mellitus (T1DM). T1DM-related chronic complications are divided into two groups based on their pathogenesis: macrovascular and microvascular. The right half of the circle shows the pathogenesis of macrovascular complications [activation of protein C kinase and direct effect of AGEs] and the list of cardiovascular complications. The left half of the circle shows the pathogenesis of microvascular complications (indirect effect of AGEs and defects in polyol metabolism) and the list of related complications. LDL; Low density lipoprotein, AGE; Advanced glycation end product, ECM; Extracellular matrix, VEGF; Vascular endothelial growth factor, GSH; Glutathione, and NADPH; Nicotinamide adenine dinucleotide phosphate.

Over the past decades new treatments such as islet cell transplantation and generating insulin producing cells from stem cell have been investigated (6-8). However, in order to discover new therapeutic approaches for T1DM, it is necessary to understand the pathophysiology of T1DM and the mechanisms of its complications. This review summarizes some of the most important genetic and environmental etiologies of T1DM and their known mechanisms of action (Fig .1) and also presents T1DMrelated chronic complications at the cellular and molecular levels (Fig .2). 

### Type 1 diabetes mellitus pathophysiology 

T1DM develops through elicitation of the immune system against beta-cell antigens and initiation of proinflammatory responses. After antigen presenting cells (APCs) present beta-cell antigens to the immune system, chronic immunological responses occur due to inefficient regulation of immunological reactions, which leads to destruction of beta-cells. Beta-cell death via virus directed or physiological mechanisms induces release of antigens and initiation of immune responses against other beta-cells. Usually dendritic cells (DCs) uptake these antigens and present them to T cells. An auto-immune response is only possible if autoreactive T cells have escaped thymic negative selection. Autoreactive T cells, activated by DCs, stimulate autoreactive cytotoxic T and B cells. Finally, effector mechanism of beta-cell destruction require the collective cooperation of DCs, macrophages, T, B, and natural killer (NK) cells (9). 

### Essential role of environmental factors in type 1 diabetes mellitus 

There are numerous environmental factors proposed to be important for development of T1DM. Some of the most cited environmental factors include reduction in gut microbiota, obesity, early introduction to fruit or cow milk during childhood, gluten, toxins, lack of vitamins, and viruses (5, 10, 11). As well, there are organs such as pancreas which take part in the pathophysiology of T1DM (Fig .1). For example, the effect of dsRNA viruses on pancreatic beta-cells and the relationship between these cells and the immune system is shown in the lower left quadrant of Figure 1. Lymph nodes and related mechanisms are presented in the upper right quadrant. 

#### Gut microbiota reduction 

Aconfrontationbetween immune cells and gut microbiota during early childhood activates immunoregulatorymechanisms which control autoimmune reactions-a phenomenon known as the "hygiene hypothesis". Toll- like receptor (TLR) 4, stimulating lipopolysaccharide (LPS), and other bacterial products that have contact with the immune system are reported as suppressors of autoimmunity (12). Therefore, a reduction in gut microbiota can lead to loss of control by the immune system, which is followed by immune cell activities against cells of the self, and finally lead to diabetes (13). 

#### Obesity 

Weight gain is another environmental issue in diabetes that results in a higher beta-cell load and increasing insulin resistance (14). The accelerator hypothesis identifies constitution, insulin resistance, and autoimmunity as accelerators of beta-cell destruction through apoptosis. However, none of the mentioned accelerators leads to diabetes without obesity (5, 15). Higher weight gain in infants has been described as a risk factor for T1DM later in childhood (16).

#### Early fruit induction

Studies show that early introduction to fruit is associated with an increase in autoimmunity to beta-cells (17-19). This association may suggest an abnormal immune response to solid food antigens in the immature gut immune system in children with HLA susceptibility to diabetes, and can take part in T1DM pathogenesis (18). Furthermore, the “overload hypothesis” suggests that environmental exposures of food may overstimulate beta-cells, thus increasing their autoimmune-mediated destruction (20). Therefore, early introduction to fruit can lead to beta-cell autoimmune-mediated destruction.

#### Early bovine milk induction 

Virtanen et al. (17) have shown that consumption of high amounts of milk products increases the risk of autoimmunity against beta-cells in young children with HLA susceptibility to diabetes. This increase may be the result of insulin autoantibody, because of the cross- reactivity between bovine and human insulin (5). Studies show that children who lack the ability to develop oral tolerance to bovine insulin are at risk for beta-cell autoimmunity. Therefore, reaction of the immune system to bovine insulin may lead to antibodies which attack human insulin in these children (5, 21).

#### Gluten

The introduction of gluten-containing foods (e.g., cereal) in diets of children younger than 3 months is associated with a significant increase in islet autoantibody production (22). Diabetic patients with human leukocyte antigen- antigen D related (HLA-DR) allele have boosted T-cell reactivity to gluten derived polypeptides. This response has been characterized by IFN-. and IL-17 secretion. Intestinal inflammation and T-cell activation induced by gluten could participate in the development of beta-cell autoimmunity (23). 

#### Toxins

Early exposure to toxins (e.g., Streptomyces-infected root vegetables) can cause an abnormal processing of proinsulin and endoplasmic reticulum stress in beta- cells of the pancreas. Exposure of the immune system to abnormal proinsulin from beta-cells may activate autoimmunity mechanisms during early life (24). 

#### Lack of vitamin D 

Epidemiological analyses present strong evidence that vitamin D decreases the risk of diabetes (25). Vitamin D can directly modify T-and B-cell functions. Vitamin D receptor (VDR) agonists induce regulatory T (Treg) cells by stimulating tolerance (26). VDR agonists stop differentiation and maturation of DCs, downregulate expression of co-stimulatory molecules such as CD40, CD80 and CD86, and reduce production of interleukin 12 (IL-12). On the other hand, VDR agonists facilitate IL10 production (27). All such mechanisms may lead to an immunosuppressive effect.

#### Viruses 

Viruses are the most researched of the mentioned environmental factors (5, 28). A variety of studies have proposed that certain viruses are linked with progression of T1DM in animal models. Human studies further showed a similar role for enteroviruses (29, 30). Viruses may lead to T1DM by at least two possible mechanisms: i. A direct cytolytic effect on beta-cells (e.g., dsRNA virus as seen in the upper left section of Figure 1) or ii. Indirect triggering of a diabetes-associated autoimmune process against beta-cells which finally leads to beta-cell destruction (e.g., viruses as seen in the upper right section of Figure 1). The latter effects of viruses are attributed to the structural similarity between some viral structures and beta-cell antigens. Persistent virus infections may also be associated with induction of autoimmunity against beta-cells. Enteroviruses, rotaviruses, cytomegalovirus, mumps virus, rubella virus, Ljungan virus, and retroviruses may be implicated in the pathogenesis of T1DM (31). 

#### Genetic factors 

Genetic studies propose a considerable heritability (more than 80%) for T1DM (32). Thus far, genome-wide association studies (GWAS) and meta-analyses have identified almost 60 genes which contribute to the genetic susceptibility to T1DM (33). These genes are expressed in different cells of the immune system or pancreatic beta-cells, which reflect the autoimmune nature of the disease. In addition to risk prediction and heritability, these genes are considered valuable clues to molecular mechanisms of T1DM. Although detailed mechanisms of T1DM are mostly unknown, here we briefly describe some mechanisms for the genetic pathogenesis of T1DM by focusing on genes with recognized mechanisms. 

#### Impaired central immune self-tolerance 

Autoimmune diseases such as T1DM are caused by failure of self-tolerance mechanisms. Genetic factors of the genomic locus of HLA are considered to account for almost half of the genetic risk of T1DM (34, 35). Therefore, genetic factors of T1DM can be categorized into HLA and non-HLA factors in terms of their impact on genetic risk of the disease. Most associations between T1DM and the HLA locus pertain to HLA class II genes. These genes are expressed in APCs such as DCs, macrophages and the thymus epithelium. In the thymus epithelium, HLA class II is responsible for presentation of self-antigens which leads to development of T cell self-tolerance. Inefficient HLA class II alleles involved in interacting and presenting insulin in thymic epithelium are relatively associated with T1DM (36). This may permit insulin- reactive T cells to escape negative selection. Lack of insulin expression in the thymus may also hamper negative selection. Polymorphisms which impair insulin gene expression in the thymus, but not beta-cells, are associated with T1DM (37, 38). 

Polymorphisms in protein tyrosine phosphatase non- receptor 22 (*PTPN22*) gene which encodes lymphocyte- specific tyrosine phosphatase (LYP) can also affect immune self-tolerance. LYP is a negative regulator of T cell receptor (TCR) signaling and a hyperactive LYP encoded by the PTPN22 risk variant that can inhibit TCR signaling during negative selection (39).

#### Impaired immune regulation and reactivity 

Pathways and genes involved in progression and regulation of the immune response may also contribute to the development of autoimmunity in T1DM. For instance, it is proposed that polymorphisms in HLA class I genes contribute to progression of the autoimmune response in the later stages of beta-cell destruction. This hypothesis is supported by findings that a HLA class I risk variant can bind to T1DM autoantigens including proinsulin epitopes (40, 41).

An association exists between polymorphisms in cytotoxic-Tlymphocyte-associated protein 4 gene (*CTLA4*) and T1DM (42). CTLA4 plays an immunoregulatory role in effector T cells by suppressing the T cell response (43). CTLA4 is crucial for proper repressive function of Tregs in mice (44). Consistent with this idea, research has shown an association between a CTLA4 susceptibility variant and the frequency of Tregs in humans (45). These and other evidences suggest that CTLA4 dampens the immune response through both effector and Treg cells (46); hence, its T1DM risk variants may hamper either or both of these mechanisms. BTB and CNC homology 1 gene (*BACH2*) expresses a transcription factor that regulates Treg activity. The T1DM risk associated variant of *BACH2* causes abnormal Tregs which can stimulate autoimmunity due to ineffective regulatory control on inflammatory responses (47).

Cytokine signals between the cells of the immune system may be influenced by a genetic background. Different IL and IL receptor genes such as *IL10, IL2,* and *IL2RA* (codes for the a subunit of the IL2 receptor) are among the genetic risk factors of T1DM (48). These cytokines usually have multiple functions in the immune system; however, the net effect of their polymorphisms may demine their impact in T1DM autoimmunity. For instance, *IL2RA* is required for both regulatory and effector T cells. The Tregs express this gene constitutively, while effector T cells only express it after their activation. A variant of IL2RA with higher expression has been shown to have a protective association with T1DM (49). Polymorphisms in interferon induced with the helicase C domain 1 gene (*IFIH1*) may provide an example for interaction between genetic and environment factors of T1DM. *IFIH1* is involved in evoking the immune response against RNA viruses. *IFIH1* variants with reduced expression have a protective association with T1DM (50). 

#### Beta-cell dysfunction and vulnerability 

A number of genes linked to diabetes are involved in beta-cell functions (51). Immune destruction of beta- cells is mediated by an extrinsic apoptotic pathway that involves FAS-mediated T cell interaction (52) along with proinflammatory cytokines such as IL-1ß and interferon gamma (IFN-γ) (53). Beta-cell sensitivity to these death signals can be influenced by the genetic background. For example, BACH2 is not only involved in regulation of the immune response, but also inhibits BIM activation and JNK1 phosphorylation via beta-cell response to proapoptotic signals. BACH2 has a crosstalk with another diabetes candidate gene *PTPN2*, which is an inhibitor of proapoptotic protein c-Jun N-terminal kinase 1 (JNK1) (54). The above mentioned apoptotic pathway is targeted with other T1DM genes such as *CTSH* (55) and *GLIS3* (56). *TNFAIP3*, another T1DM gene, has been shown to provide a negative feedback loop for the proapoptotic activity of nuclear factor kappa-light-chain-enhancer of activated B cells (NF-κB) (57, 58). Since nitric oxide and FAS-mediated pathways are downstream of NF-κB in beta-cells (58), impaired TNFAIP3 function may influence these inflammatory and apoptotic mechanisms. 

Most mechanisms that underlie the progression of T1DM by genetic factors remain to be determined. However, the above examples show how the genetic background can contribute to T1DM pathogenesis. Further functional analyses of these genes may shed light on the molecular mechanisms behind T1DM onset and progression. 

#### Complications 

The two major classes of late complications attributed to T1DM, microvascular and macrovascular, affect the heart, limbs, nervous system, eyes, and kidneys (Fig .2). The right half of the circle presents macrovascular complications whereas the left half shows microvascular complications. The pathogenesis of macrovascular complications is demonstrated by the role played by large vessels, the extracellular matrix (ECM), and cells in the right half of the figure. Intracellular mechanisms of neurological and lower extremity complications are shown in a neuron cell at the lower left quadrant of the circle. Finally, the upper left quadrant of the circle shows related mechanisms of ophthalmologic and renal complications. 

#### Macrovascular complications of type 1 diabetes mellitus 

Macrovascular complications comprise a group of large blood vessel diseases that occur in diabetic patients. In comparison with non-diabetics, the risk of cardiovascular disease in diabetic patients is four times higher. Coronary artery, cerebrovascular, and peripheral vascular diseases are categorized as macrovascular complications. Hemodynamic (blood pressure), metabolic (lipids and glucose), and genetic factors can increase the risk of these complications. Hyperglycemia is a major biochemical factor that increases the probability of cardiovascular disease. In addition, hypertension can increase the risk of diabetic related macrovascular complications such as coronary artery disease and stroke. Risk of hypertension in T1DM patients is 30% higher than non-diabetics. Oxidative stress plays an important role in hypertension related damage to vascular endothelial cells and cardiac hypertrophy. Optimal blood glucose and hypertension control in diabetics are effective ways to reduce the risk of macrovascular complications (59, 60). 

#### Microvascular complication of type 1 diabetes mellitus

Damage to small vessels (capillaries) during high blood glucose levels can cause microvascular complications in tissues where glucose uptake is independent of insulin such as with neurons, the kidneys, and retina. Hyperglycemia, as the most important risk factor in diabetics, can cause neuropathy, nephropathy, and retinopathy by different mechanisms. Some of these mechanisms are more important in specific complications. Here, we classify microvascular complications into three categories–retinopathy, neuropathy, and nephropathy (60). 

#### Retinopathy 

Diabetes related damage to the macula, retina, or both can cause visual problems and blindness. The probability of retinopathy as a common diabetic complication is closely related to the duration of diabetes. Up to 50% of T1DM patients are at risk for retinopathy. Microvascular changes in diabetics as a result of hyperglycemia such as small vessel basement membrane thickening and increase in endothelial cell permeability can cause ophthalmological and renal complications (61). 

#### Neuropathy

Damage from hyperglycemia to peripheral nerves, including sensory, autonomic, and motor neurons, can cause neuropathy. Hyperglycemia, disease duration, and genetic factors can increase the risk of this complication. Peripheral neuropathy can be characterized by axonal thickening, axonal loss, loss of microfilaments, neural demyelination, and neural death (61). 

#### Nephropathy 

Diabetic nephropathy is characterized by loss of glomerular filtration rate, albuminuria (>300 mg/day), and damage to glumeruli. Diabetic nephropathy can be seen in about 30-40% of diabetics. Hyperglycemia, hypertension, and hyperlipidemia are the main metabolic risk factors that increase kidney disease by several known metabolic pathways (61).

### Pathophysiology of macro-and microvascular complications

Several mechanisms have a role in the pathogenesis of micro- and macrovascular complications. We classify these mechanisms into the following four categories (61). 

### Direct effect of advanced glycation end products

During long-standing hyperglycemia in diabetics, glucose forms covalent bonds with proteins through a non-enzymatic reaction between the free amino group of an amino acid and the carbonyl group of reducing sugars. This process leads to formation of advanced glycation end products (AGEs). Glycation disrupts molecular conformation and alters protein function. AGEs have crucial role in diabetes related cardiovascular and renal complications (62). AGEs can bind to intracellular and extracellular proteins and alter tissue functions. Binding of AGES to ECM proteins creates anchoring sites for proteins such as albumin, collagen, and elastin that leads to ECM thickening and atherosclerosis. Interactions of AGEs with ECM can impair matrix-cell and matrix- matrix interactions. This can induce cell death, cell differentiation, and cell migration. In cardiomyocytes, interaction of AGEs with intracellular proteins such as Ryanodine can disrupt Ca^2+^ homeostasis and induce the risk of heart related complications. Diabetic patients with cardiovascular disease have higher than normal serum AGEs. The high level of AGEs in serum can be used as a biomarker for cardiovascular diseases (63).

### Indirect effect of advanced glycation end products

Binding of AGE to the cell’s surface receptor leads to activation of multiple signaling pathways inside the cells and different responses of endothelial cells, smooth muscle cells, macrophages, and Tcells. Activation of nicotinamide adenine dinucleotide phosphate (NADPH) and the MAPK pathway in response to AGE interaction with cell surface receptors can induce reactive oxygen species (ROS) production and NFĸB activation, respectively. ROS has pivotal roles in diabetes related cardiovascular and ophthalmological complications. Transcription activation of multiple genes such as IL-6, tumor necrosis alpha (TNF-α), and vascular endothelial growth factor (VEGF) by NFĸB can increase inflammation and arthrosclerosis (63). In different cell types, an increase in pre-clotting activity occurs in response to AGE interactions with cell surface receptors. In addition to pre-clotting activity cytokine production in T cells and macrophages, there is an increase in the dividing rate in smooth muscles and stimulation of ECM secretion by these cells can be seen during AGEs interactions with their related receptors (64). 

### Activation of protein kinase C

Diacylglycerol (DAG) accumulation in cells as a result of hyperglycemia can induce protein kinase C (PKC) activation. PKC is a type of serine/threonine kinase that has multiple isoforms. Different isoforms of this enzyme can be activated in various tissues to induce different complications. Hyperglycemia can induce ß and d isoform activation in vascular cells (65). The DAG-PKC pathway can induce cardiovascular complication by multiple ways such as ECM synthesis, angiogenesis and change of vascular permeability by VEGF production, cytokine activation, and cell growth. PKC ß overexpression in transgenic mice can cause cardiomyopathy. In addition to activation of ROS and inflammation in cardiomyocytes in response to PKC activation, PKC can induce insulin resistance by phosphorylation of serine/threonine residues in cardiomyocytes. Disruption of insulin metabolism in cardiac cells can induce heart related complications (66). 

### Defects in polyol metabolism

In hyperglycemia, disruption of normal glucose metabolism leads to activation of the polyol pathway. Polyol pathway activation can cause peripheral nerve damage and increase the risk of lower limb amputation, or neuropathy (67). In hyperglycemia, there is a decrease in the level of glutathione (GSH) which is a precursor of NADPH. Decreased NADPH causes less production of fructose from sorbitol. The polyol pathway in neurons can cause cell death by osmotic damage and ROS production. In addition to the polyol pathway, PKC, AGEs, and hexosamine pathways have important roles in diabetes related neuropathy. These pathways can induce ROS and inflammation in neurons. Among the all above mentioned mechanisms, PKC activation and direct/indirect effects of AGE play a role in vascularization which has a critical role in numerous T1DM complications (68)

However, environmental factors are not the only pathogenic source of complications. Genetic factors can also affect this process. GWAS have important roles in discovering diabetes complication related genes and pathways. Identification of complication specific genetic variants can facilitate improvement of new and targeted therapeutic methods for each specific diabetes related complication. Different genetic variants have been discovered for diabetes complications. Diabetic vascular complication is good example that clarifies the role of genetic factors, environmental factors, and their interactions in disease progression. Polymorphism in lipid related metabolism genes such as APOE, APOB, APOC, CETP, and PON increase the risk of macrovascular complications in diabetic patients compared to healthy individuals. In addition to diabetic related cardiovascular complications, the role of genetic factors in diabetic related retinopathy, nephropathy, and neuropathy have been studied. VEGFA (encodes vascular endothelial growth factor A), and AKR1B1 (encodes aldose reductase, one of the polyol pathway enzymes) are the two best studied genes that play a role in diabetic related retinopathy. A study of the diabetics genome revealed that 11 single nucleotide polymorphism (SNPs) in different chromosomes could increase the risk of nephropathy. SNPs on chromosomes 7p, 9p, and 11p that are located near CPVL, FRMD3, and CARS play important roles in induction of nephropathy risk (69, 70). 

## Conclusion

This review has discussed T1DM pathogenesis, the role of primary genetic and environmental factors in this process, and the mechanisms of complications. However, much remains to be understood. Therefore, research efforts to elucidate the underlying mechanisms of T1DM can provide further therapeutic options for T1DM treatment. 
